# A Novel Rules Based Approach for Estimating Software Birthmark

**DOI:** 10.1155/2015/579390

**Published:** 2015-04-06

**Authors:** Shah Nazir, Sara Shahzad, Sher Afzal Khan, Norma Binti Alias, Sajid Anwar

**Affiliations:** ^1^Department of Computer Science, University of Peshawar, Peshawar 25000, Pakistan; ^2^Department of Computer Science, Abdul Wali Khan University, Mardan 23200, Pakistan; ^3^Ibnu Sina Institute for Fundamental Science Studies, Universiti Teknologi Malaysia, 81310 Johor Bahru, Malaysia; ^4^Institute of Management Sciences, Peshawar 25000, Pakistan

## Abstract

Software birthmark is a unique quality of software to detect software theft. Comparing birthmarks of software can tell us whether a program or software is a copy of another. Software theft and piracy are rapidly increasing problems of copying, stealing, and misusing the software without proper permission, as mentioned in the desired license agreement. The estimation of birthmark can play a key role in understanding the effectiveness of a birthmark. In this paper, a new technique is presented to evaluate and estimate software birthmark based on the two most sought-after properties of birthmarks, that is, credibility and resilience. For this purpose, the concept of soft computing such as probabilistic and fuzzy computing has been taken into account and fuzzy logic is used to estimate properties of birthmark. The proposed fuzzy rule based technique is validated through a case study and the results show that the technique is successful in assessing the specified properties of the birthmark, its resilience and credibility. This, in turn, shows how much effort will be required to detect the originality of the software based on its birthmark.

## 1. Introduction

A software birthmark is an intrinsic property of software that is used to detect the theft of software systems. A software system can be stolen or pirated which ultimately results in financial loss to the owner organization. Software piracy is a global problem of unauthorized copying, installing, using, distribution, or sale of software other than what is officially documented as exclusive rights by the authors as described in relevant license agreement. With the growth of software development industry, manufacturing and use of Internet and software piracy have become a red alert sign for numerous software industries. Software companies encounter tremendous losses due to software piracy. On the other hand, software pirates earn huge sums of money from the piracy which they are doing. General international community is not yet aware of the serious crime that is being done. Software piracy happens in diverse ways which includes hard-disk loading, soft lifting, counterfeit goods, rental software, and bulletin board piracy [1]. Original licensed software has many advantages. These advantages include assurance of the disk carrying no virus, regular software upgrades, consistent technical support, complete documentation, and quality assurance.

Different advanced techniques are used for the detection and prevention of software theft, such as software watermarking and software fingerprints [2–9]. Software watermarks emphasize ownership of program, while fingerprint is used in tracking the intellectual property. Besides these techniques, software birthmark is a property based system which identifies the inherent property of a program to check and show the originality of software. Most of the study on software birthmark focuses on how to describe the appropriate properties to detect software theft.

The contribution of this paper is to estimate software birthmark to show its effectiveness. The estimation is based on the well-defined properties of birthmarks. The method provides an intelligent solution to estimate two commonly used properties that are credibility and resilience which in turn will provide estimates of the birthmark. For this purpose, a fuzzy model has been designed which is based on membership function and fuzzy rules to provide an appropriate estimation for software birthmarks.

The structure of the rest of the paper is as follows. In Section 2, research on software birthmark has been discussed.  Section 3 gives a detailed description of materials and method. In this section, the details of software birthmark, classifications of birthmarks, and a comparison with watermarks are given. The proposed methodology for estimating software birthmarks is presented in Section 4. Results and discussion of the method are presented in Section 5. The paper is concluded in Section 6.

## 2. Related Work

Till now, researchers have considered two important properties of a software birthmark to evaluate their effectiveness that are credibility and resilience. Zeng et al. [10] report that not many theoretical frameworks are available that properly analyze and verify the success of software birthmark. The evaluation of software birthmark is mainly done through experiment. They presented a semantic based abstract interpretation framework. This model describes two important properties of software birthmark, namely, credibility and resilience. With the help of static *n*-gram birthmark and static API birthmark, the effectiveness of the framework is verified. Myles and Collberg [11] presented a technique called “Whole Program Path Birthmarking” for detecting software theft. The program is based on complete control flow of the software program. The two important properties that are credibility and tolerance are considered to evaluate the efficiency of the technique. The technique demonstrates that the whole program path birthmark is more resilient than the existing techniques. Furthermore, the technique also showed that even if an embedded watermark is destroyed by program transformation, the birthmark can still identify the theft. Park et al. [8] used static API trace birthmark for the detection of Java theft. The method evaluates the birthmark in terms of credibility and resilience. The experimental result of the method shows that static API birthmark can detect modules of two packages whereas the other birthmark fails.

Kakimoto et al. [12] analyzed the birthmark similarities in ArgoUML and visualized them using multidimensional scaling. Chan et al. [13] proposed a dynamic software birthmark system for systems designed in Java based on the object reference graph. The method was evaluated for huge programs and most of them were megabytes in size. The results showed that the method was useful in detecting the code theft. Wang et al. [14] used CHI (*χ* ^2^ statistics) for the characteristics selection in text classification and brought in an instruction words software birthmark selection. The algorithm makes sample program for protected program and takes out instruction word from sample program according to instruction word library. To find out their correlation, the *χ* ^2^ statistics is calculated for each instruction word and program. The experimental results of the algorithm show that selection algorithm has much enhanced the robustness and credibility of the birthmark. Choi et al. [6] proposed a static API software birthmark for Windows binary executable. They compared 49 Windows executables and showed that their birthmark can differentiate and detect the copies. The birthmark is compared with the Windows dynamic birthmark and showed that it is more suitable for GUI application. Lim [15] presented a customized method of *k*-gram birthmark which permits the small changes of programs by applying partial matching of *k*-gram. The experimental result shows that customizing the *k*-gram birthmark improves the properties of birthmark that are credibility and resilience. The idea of rule based estimation has been used by Tyagi and Sharma [16]. They measured the reliability of component based system. Fuzzy rules were designed to measure the reliability based on four factors that are application complexity, reusability, component dependency, and operational profile.

## 3. Materials and Method

The following are the main concepts used to define the proposed birthmark estimation technique.

### 3.1. Software Piracy

**Figure 1:**
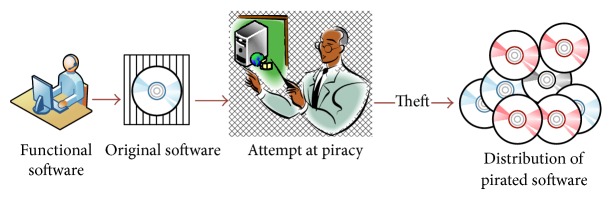
Software piracy.

The software industry has faced huge financial losses due to the piracy of software. Software piracy is performed by end-users as well as the dealers. Software piracy causes serious problems which hinder the success of the international software industry. Piracy of software is a global problem of illegal copying, installation, use, distribution, or sale of software in any manner other than that expressed in the appropriate license agreement. The pirates gain easy benefits from the sale of pirated software which ultimately affects the business of the software industry.  Figure 1 shows how software is pirated from its original business market.

The original licensed software offers a number of high valued benefits to the customers, including assurance of software quality, availability of upgrades, technical and manual documentations, and less bandwidth consumption. On the other hand, pirated software does not provide such kind of facilities. If an organization is using pirated software, there might be risk of failure of the system, which might put the organization at risk of huge financial loss.

### 3.2. Software Birthmark

Software birthmark is a unique property of every type of software which can help in detecting software theft. It is the intrinsic characteristics of a program or software that can be used to spot the theft. Comparing the birthmarks of software tells us whether a program or software is a copy of any other software or not. The following definitions of birthmark are given by Tamada et al. [17].

**Definition 1 (birthmark).**

Suppose *p* and *q* are the programs. Let *f* be the function for extracting a set of properties from a program. *f*(*p*) is a birthmark of *p* if and only if

1. *f*(*p*) is obtained only from *p* itself;
2. *q* is a copy of *p*⇒*f*(*p*) = *f*(*q*).

**Definition 2 (dynamic birthmark).**

Suppose *p* and *q* are the programs. Input *i* is given to these programs. Let *f* be extracting characteristics from a program. So *f*(*p*, *i*) is a dynamic birthmark of *p* if and only if

1. *f*(*p*, *i*) is obtained only from *p* itself by extracting *p* with the given input *i*;
2. *q* is a copy of *p*⇒*f*(*p*, *i*) = *f*(*q*, *i*).

All program paths cannot be covered by the dynamic birthmarks; dynamic birthmark only detects the theft of the program. On the other hand, static birthmark is extracted by the static program analysis, that is, liable to the properties of overestimated program.

### 3.3. Classification of Software Birthmark

Software birthmark is classified into the following three categories [10].

*(i) Instruction Based Software Birthmarks.* Software program consists of data and instructions. Instruction sequences can reflect program behavior to various points, so it is realistic to define birthmarks as instructions sequences.

*(ii) API Based Software Birthmark.* Several birthmarks are available that are based on observations of the way a program uses the standard API libraries. Not only is it unique to a program, but this feature is also complex for an attacker to forge [18].

*(iii) Graph Based Software Birthmark.* The software program is like graph structure. For instance, functions are represented as control flow graph, dependency among statement(s) in the function(s) is represented as dependency graph, possible calls between functions are represented as call graph, and inheritance interaction between classes is represented as acyclic graph. As a result, it makes sense to represent a birthmark using graph representations of programs [18].

### 3.4. Birthmark and Watermark

Software birthmark is a promising technique used for the detection of software theft. Birthmark does not embed additional code or information in any form in the original program. Software birthmarks only extract the inherent characteristics from the original program to detect the originality of program [11]. Software birthmark only establishes an identity to detect if a program is a copy of any other program. It does not show who the original owner of the program is or who is guilty of software piracy [11]. While software watermarking asserts the ownership of the programs by adding extra information to the original program before it is publically available, software watermarks identify software from the embedded information/code. Both the techniques can be combined to provide a stronger verification mechanism to detect theft. Birthmark can be used where there is a limitation of storage space as watermarking uses extra storage space. Also, in many situations, watermarks fail, for example, if an attacker is able to apply obfuscation that destroys watermarks. In such situations, software birthmarks provide evidence of piracy or software theft [11].

## 4. Proposed Methodology

The following sections define the proposed methodology to estimate software birthmarks.

### 4.1. Software Birthmarks Properties

In order to estimate the success of software birthmarks, researchers typically consider two properties, which are credibility and resilience [19]. Credibility requires that the birthmark of the two programs must be different, whereas the resilience states that the birthmark should be preserved and not destroyed in any circumstances.

According to Tamada et al. [17] software birthmark satisfies the following two important properties which indicates that the two independently implemented programs should be different.

**Property 1 .**

Let *P* and *Q* be two independently written programs which achieve the same task; then *f* is credible if *f*(*P*) ≠ *f*(*Q*).

**Property 2 .**

Let *P*′ be the program obtained from *P* by applying semantic preserving transformation *T*. *f* is resilient to *T* if *f*(*P*) = *f*(*P*′).

Property 1 indicates that the birthmarks falsely show that *Q* is a copy of *P*. This situation will occur with the separately implemented programs that achieve the same task.

Property 2 relates to identifying a copy in the occurrence of transformation. It is wished that a birthmark could be used to detect a copy if some transformation has been applied to the program.

**Figure 2:**
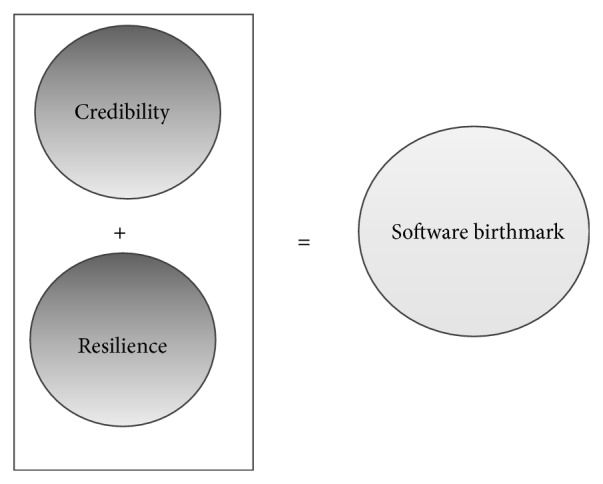
Properties based software birthmarks.

Figure 2 shows the properties of software birthmark.

In the existing literature on software birthmarks, there is no model which exactly estimates the birthmark of software based on the properties of credibility and resilience. The proposed methodology helps to estimate the birthmarks of software based on these properties.

### 4.2. Fuzzy Logic

Fuzzy logic concept was developed by Zadeh in 1965 [20]. It is a mathematical tool which deals with managing uncertain and doubtful information. Fuzzy set theory is being used for solving diverse problems in different fields of daily life. Fuzzy tool helps in providing solution for the problems which are complicated to model. Fuzzy set is the extended form of traditional sets, which is described by membership function and is extremely beneficial for decision making in uncertain and vague situations. Here, the decision can be made in qualitative variables (low, strong, very strong, etc.) instead of quantitative variables (i.e., numbers), and these qualitative variables allow precise modeling. The inputs and outputs have the degree of membership function in range of interval [0, 1].

In the proposed method, the membership functions named mf_1_ in the range of (0–19), mf_2_ in the range of (20–39), mf_3_ in the range of (40–59), mf_4_ in the range of (60–79), and mf_5_ in the range of (80–100) are defined. Also, to plot fuzziness triangular membership functions are defined and used to represent weights. The triangular membership function has three parameters (*l*, *m*, *u*), which are defined as *l* ≤ *m* ≤ *u*.

Details of fuzzy logic concept are given in Zadeh [20]; however the major parts of the fuzzy system are as follows. The first phase is the fuzzification, which transforms the classification table into continuous classifications. Then, it is processed in the fuzzy domain based on the designed rules. Lastly, the fuzzification process transforms fuzzy number back into the real number.

### 4.3. Rules Based Approach to Estimate Software Birthmark

Estimating software birthmark is an essential part of software system development to get rid of the entire theft of the software system. Most of software theft threats are faced during the implementation of the software. Developers are still in confusion about how to handle such situations. If birthmarks of the system are estimated, then one can easily make decision about the alternate design. The proposed methodology, based on fuzzy concept, provides an estimation model to software birthmark. Initially inputs (properties of birthmark) are selected on the basis of which the birthmark(s) is to be estimated. On the basis of inputs, the membership functions are plotted. The membership function identifies the degree of relationship of the concept (data) to a particular area (data range). Five membership functions were plotted that are mf_1_, mf_2_, mf_3_, mf_4_, and mf_5_. The inputs and membership functions are combined in rule editor which forms fuzzy rules. A fuzzy inference system model is obtained based on membership functions and rules.

#### 4.3.1. Algorithm for Designing a Rule Based Model

The following are the steps to design the proposed model.

1. Perform domain analysis on software birthmark.
2. Identify properties of software birthmark on which birthmark is to be estimated.
3. Establish an input data base for these properties.
4. Design the fuzzy inference system based on these properties (inputs).
5. Define the membership functions for these properties (for both inputs and output).
6. Design the fuzzy rules based on membership functions.
7. Obtain a fuzzy inference system (model to estimate birthmark).
8. Estimate the inputs accordingly.

**Figure 3:**
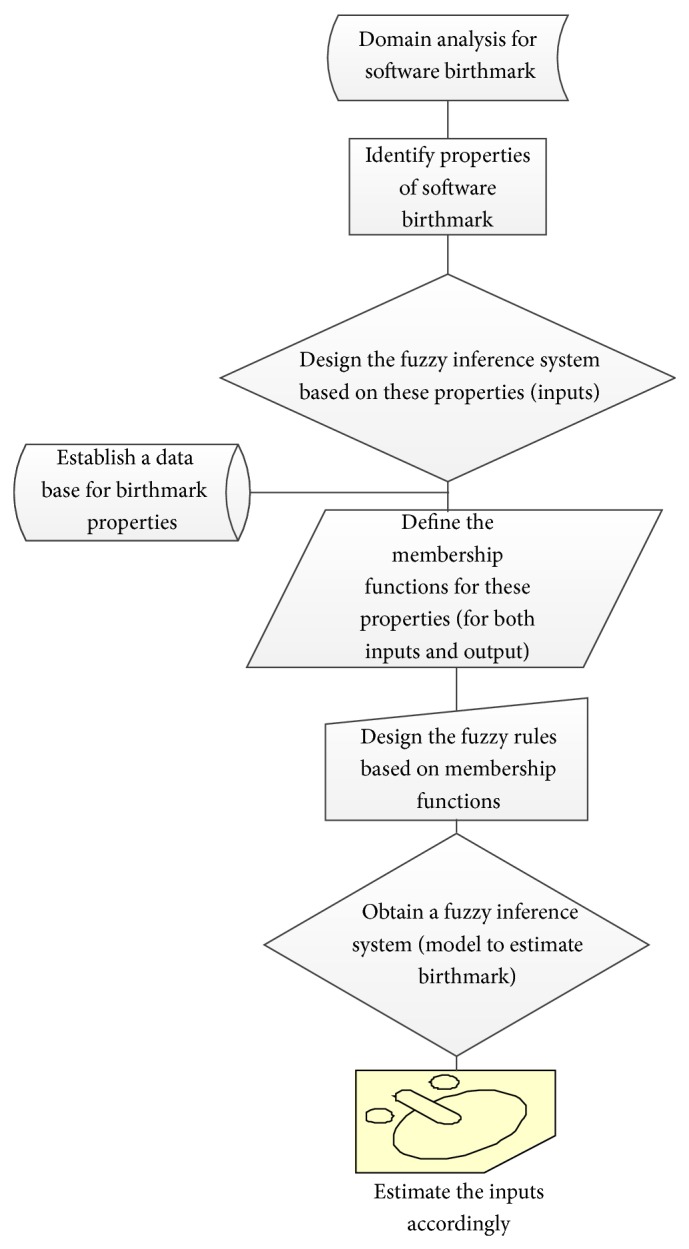
Graphical representation of the proposed algorithm.

The graphical representation of the algorithm is given in Figure 3.

The proposed work for estimating software birthmark has been carried out by using MATLAB fuzzy tool box [21].

The different membership combinations are given in Table 1.

**Figure 4:**
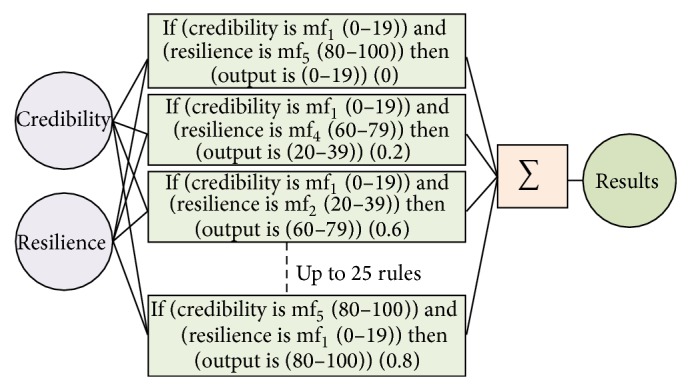
Proposed fuzzy rules model.

The fuzzy rules and model in the proposed methodology are given in Figure 4.

**Figure 5:**
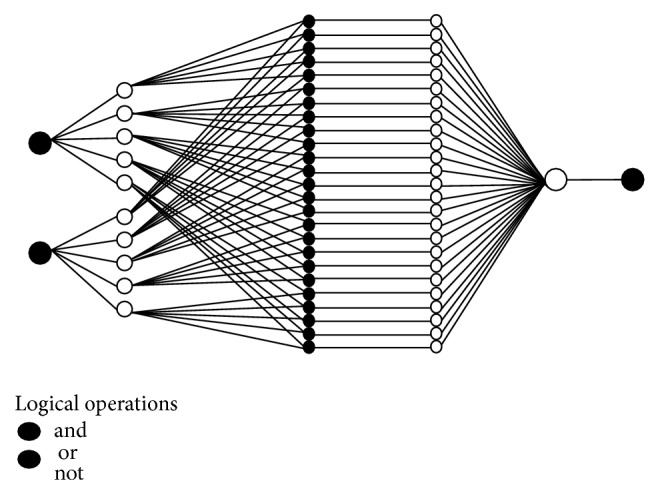
Detailed fuzzy rules model (inputs, membership functions, rules, and output).

The proposed model can further be explicitly explained in Figure 5.

The rules are as follows.

 If (credibility is mf_1_(0–19)) and (resilience is mf_5_(80–100)) then (output is (0–19))  (0).
 If (credibility is mf_1_(0–19)) and (resilience is mf_4_(60–79)) then (output is (20–39)) (0.2).
 If (credibility is mf_1_(0–19)) and (resilience is mf_3_(40–59)) then (output is (40–59)) (0.4).
 If (credibility is mf_1_(0–19)) and (resilience is mf_2_(20–39)) then (output is (60–79)) (0.6).
 If (credibility is mf_1_(0–19)) and (resilience is mf_1_(0–19)) then (output is (80–100)) (0.8).
 If (credibility is mf_5_(80–100)) and (resilience is mf_1_(0–19)) then (output is (80–100)) (0.8).
 If (credibility is mf_4_(60–79)) and (resilience is mf_1_(0–19)) then (output is (60–79)) (0.6).
 If (credibility is mf_3_(40–59)) and (resilience is mf_1_(0–19)) then (output is (40–59)) (0.4).
 If (credibility is mf_2_(20–39)) and (resilience is mf_1_(0–19)) then (output is (20–39)) (0.2).
 If (credibility is mf_2_(20–39)) and (resilience is mf_2_(20–39)) then (output is (80–100)) (0.8).
 If (credibility is mf_3_(40–59)) and (resilience is mf_3_(40–59)) then (output is (80–100)) (0.8).
 If (credibility is mf_4_(60–79)) and (resilience is mf_4_(60–79)) then (output is (80–100)) (0.8).
 If (credibility is mf_5_(80–100)) and (resilience is mf_5_(80–100)) then (output is (80–100)) (0.8).
 If (credibility is mf_2_(20–39)) and (resilience is mf_5_(80–100)) then (output is (20–39)) (0.2).
 If (credibility is mf_3_(40–59)) and (resilience is mf_5_(80–100)) then (output is (40–59)) (0.4).
 If (credibility is mf_4_(60–79)) and (resilience is mf_5_(80–100)) then (output is (60–79)) (0.6).
 If (credibility is mf_3_(40–59)) and (resilience is mf_4_(60–79)) then (output is (60–79)) (0.6).
 If (credibility is mf_2_(20–39)) and (resilience is mf_4_(60–79)) then (output is (40–59)) (0.4).
 If (credibility is mf_2_(20–39)) and (resilience is mf_3_(40–59)) then (output is (40–59)) (0.4).
 If (credibility is mf_4_(60–79)) and (resilience is mf_3_(40–59)) then (output is (60–79)) (0.6).
 If (credibility is mf_5_(80–100)) and (resilience is mf_3_(40–59)) then (output is (80–100)) (0.8).
 If (credibility is mf_4_(60–79)) and (resilience is mf_2_(20–39)) then (output is (60–79)) (0.6).
 If (credibility is mf_3_(40–59)) and (resilience is mf_2_(20–39)) then (output is (40–59)) (0.4).

**Figure 6:**
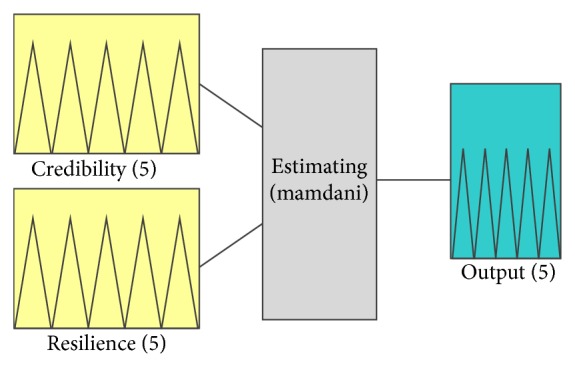
Proposed fuzzy inference system.

Based upon the above rules, a fuzzy inference system is obtained for estimating software birthmark, which is given in Figure 6.

**Figure 7:**
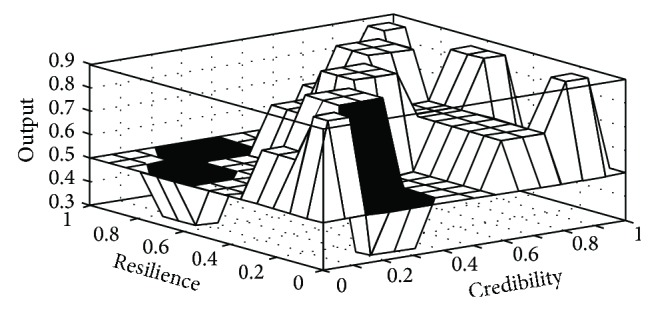
Surface view of inputs and outputs (generated in MATLAB).

Figure 7 visually shows the surface view of inputs and output.

### 4.4. Inputs Estimation

Once the fuzzy rules model is designed, inputs will be given according to the customer requirements to the model. The model will generate the output based on the fuzzy rules. Details of the proposed system, inputs, and output are given as shown in Table 2.

### 4.5. Evaluation of the Model (Case Study)

The present research work has been validated by a case study of small module for Android application. The Android radiocalc module consists of 109 lines of code. The methodology has been applied on a similar application for Android. The birthmark of the module has been estimated based on the properties of resilience and credibility.

*K*-gram based birthmark similarity technique [22] has been used. By performing various experiments we found out that as the *K*-value increases the birthmark similarity decreases. For very small values of *K* the birthmark similarity was not satisfactory. For *k* = 5, the experiment revealed good results in terms of similarity and runtime overhead. The resulting similarity for the above mentioned application with *k* = 5 was 40%.

We applied SandMark [23] and Codeshield [24] tools for the above application for code obfuscation. To find the value of resilience, it gives a similarity of 80% for *k* = 5. Codeshield tool provides the name obfuscation, the removal of debugging information, and some type of control flow, while the SandMark tool does not include an automatic obfuscation. The similarity was computed through *K*-grams. The similarity of Codeshield was found for *K*-gram, which shows that if *K* increases, there is a decrease in the similarity for numerous transformations.  Table 3 shows the inputs and values for the proposed model.

The defined inputs to the fuzzy model are described as follows. If credibility is equal to 0.4 (40%) and resilience is 0.8 (80%), these inputs are given to the fuzzification model (fuzzy inference system). Credibility 0.4 is the degree of membership function mf_1_ (40–59) and resilience 0.8 is the degree of membership function mf_2_ (20–39). It will give the output 0.500 from the degree of membership function based on the designed model. So from the results one can make a decision about the birthmark of the software.

## 5. Results and Discussion

A fuzzy inference system is designed which models the system which in turn estimates the birthmark of the software. Inputs are assigned to the model to check and estimate the software birthmark in terms of credibility and resilience. The designed model evaluates the inputs (which are given to the model) and gives results. On the basis of the given results, one can check the estimation of software birthmark for the properties of credibility and resilience. To check the validity of the proposed model, inputs were given as follows: out = evalfis $(\left[\begin{array}{cc} 0.4 & 0.8 \end{array}\right]$, fismat) and the output = 0.500, which show the estimation of the software birthmark. Hence, this result clearly shows the software birthmarks for their desired properties. Different fuzzy techniques are used [25], which use fuzzy C-mean clustering.

## 6. Conclusion

Software theft is a global problem of copying, stealing, and misusing the software without proper license agreement. Software birthmark is a capable technique to detect the theft of software systems. Software birthmark is an intrinsic characteristic of software used to detect the similarity of software. The estimation of software birthmark can play a key role in accepting the effectiveness of a birthmark. In this research, fuzzy logic has been used to estimate software birthmark(s), which is an efficient and powerful tool to tackle issues of uncertainty. This method is based on fuzzy rules which were designed from the fuzzy membership functions. Different techniques are used in practice but all are based on known information. In practice situations of uncertainty also arise. The proposed model works well in case of uncertainty and with unknown information. The model is based on the two properties of software birthmark, credibility and resilience. The model has been validated using some Android applications. Various experiments have been performed using different existing tools of code obfuscation and software birthmark(s) are estimated. Results produced by the proposed process show that the method is efficient and provides satisfactory results. The approach has been tested only for credibility and resilience as these two properties are considered as the most important properties of software birthmark(s). Therefore, these are selected here for model testing. In the future, the model can be expanded for a different set of properties.

## Figures and Tables

**Table 1:** Membership function pairs.

mf_1_, mf_1_	mf_1_, mf_2_	mf_1_, mf_3_	mf_1_, mf_4_	mf_1_, mf_5_

mf_2_, mf_1_	mf_2_, mf_2_	mf_2_, mf_3_	mf_2_, mf_4_	mf_2_, mf_5_

mf_3_, mf_1_	mf_3_, mf_2_	mf_3_, mf_3_	mf_3_, mf_4_	mf_3_, mf_5_

Mf_4_, mf_1_	mf_4_, mf_2_	mf_4_, mf_3_	mf_4_, mf_4_	mf_4_, mf_5_

mf_5_, mf_1_	mf_5_, mf_2_	mf_5_, mf_3_	mf_5_, mf_4_	mf_5_, mf_5_

**Table 2:** Proposed model (inputs and output).

Model	[System] Name = “estimating” Type = “mamdani” Version = 2.0 NumInputs = 2 Num Outputs = 1 And Method = min Or Method = max Imp Method = min Agg Method = max Defuzz Method = centroid

[Input1]	Name = “Credibility” Range = $\left[\begin{array}{cc} 0 & 1 \end{array}\right]$ Num MFs = 5 MF_1_ = mf_1_(0–19) trimf, $\left[\begin{array}{ccc} 0 & 0.1 & 0.19 \end{array}\right]$ MF_2_ = mf_2_(20–39) trimf, $\left[\begin{array}{ccc} 0.2 & 0.3 & 0.39 \end{array}\right]$ MF_3_ = mf_3_(40–59) trimf, $\left[\begin{array}{ccc} 0.4 & 0.5 & 0.59 \end{array}\right]$ MF_4_ = mf_4_(60–79) trimf, $\left[\begin{array}{ccc} 0.6 & 0.7 & 0.79 \end{array}\right]$ MF_5_ = mf_5_(80–100) trimf, $\left[\begin{array}{ccc} 0.8 & 0.9 & 1 \end{array}\right]$

[Input2]	Name = “Resilience” Range = $\left[\begin{array}{cc} 0 & 1 \end{array}\right]$ Num MFs = 5 MF_1_ = mf_1_(0–19) trimf, $\left[\begin{array}{ccc} 0 & 0.1 & 0.19 \end{array}\right]$ MF_2_ = mf_2_(20–39) trimf, $\left[\begin{array}{ccc} 0.2 & 0.3 & 0.39 \end{array}\right]$ MF_3_ = mf_3_(40–59) trimf, $\left[\begin{array}{ccc} 0.4 & 0.5 & 0.59 \end{array}\right]$ MF_4_ = mf_4_(60–79) trimf, $\left[\begin{array}{ccc} 0.6 & 0.7 & 0.79 \end{array}\right]$ MF_5_ = mf_5_(80–100) trimf, $\left[\begin{array}{ccc} 0.8 & 0.9 & 1 \end{array}\right]$

[Output]	Name = “output” Range = $\left[\begin{array}{cc} 0 & 1 \end{array}\right]$ Num MFs = 5 MF_1_ = (0–19) trimf, $\left[\begin{array}{ccc} 0 & 0.1 & 0.19 \end{array}\right]$ MF_2_ = (20–39) trimf, $\left[\begin{array}{ccc} 0.2 & 0.3 & 0.39 \end{array}\right]$ MF_3_ = (40–59) trimf, $\left[\begin{array}{ccc} 0.4 & 0.5 & 0.59 \end{array}\right]$ MF_4_ = (60–79) trimf, $\left[\begin{array}{ccc} 0.6 & 0.7 & 0.79 \end{array}\right]$ MF_5_ = (80–100) trimf, $\left[\begin{array}{ccc} 0.8 & 0.9 & 1 \end{array}\right]$

**Table 3:** Inputs and value for the proposed model.

Inputs	For *k* = 5	
	Value in %	Value for proposed model

Credibility	40%	0.4
Resilience	80%	0.8
